# Supplementary material for: Prevention and therapy of SARS-CoV-2 and the B.1.351 variant in mice
Source: Cell Rep. Author manuscript; Available in PMC 2021 Aug 16. (PMC8270748; doi:10.1016/j.celrep.2021.109450)
Supplement: 1 [file NIHMS1728470-supplement-1.pdf]

Cell Reports, Volume *36*

Supplemental information

Prevention and therapy of SARS-CoV-2 and the B.1.351 variant in mice

David R. Martinez, Alexandra Schäfer, Sarah R. Leist, Dapeng Li, Kendra Gully, Boyd Yount, Joy Y. Feng, Elaine Bunyan, Danielle P. Porter, Tomas Cihlar, Stephanie A. Montgomery, Barton F. Haynes, Ralph S. Baric, Michel C. Nussenzweig, and Timothy P. Sheahan

Figure S1

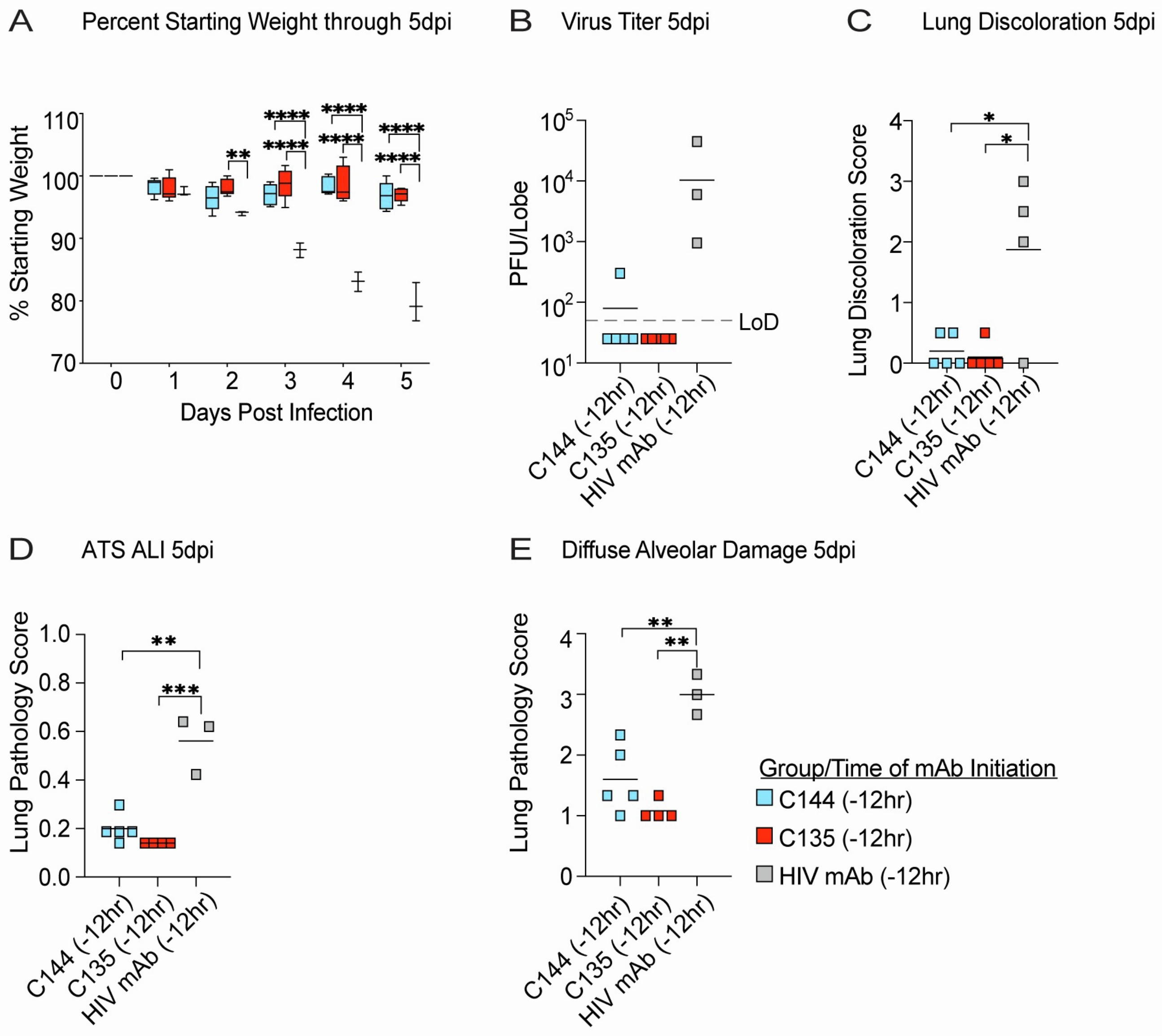

Figure S1. The prophylactic efficacy of mAb monotherapy against SARS-CoV-2 in mice treated at 12 hours before infection.

(A) % starting weight in therapeutically treated mice with C144, C135, or an HIV mAb at 12 hours before infection.

(B) Lung viral titers in therapeutically treated mice at 12 hours before infection.

(C) Lung discoloration score in therapeutically treated mice at 12 hours before infection.

(D-E) Lung pathology in therapeutically treated mice at 12 hours before infection. P values are from a 2-way ANOVA after Sidak's multiple comparisons test. Related to Figure 3.

Figure S2

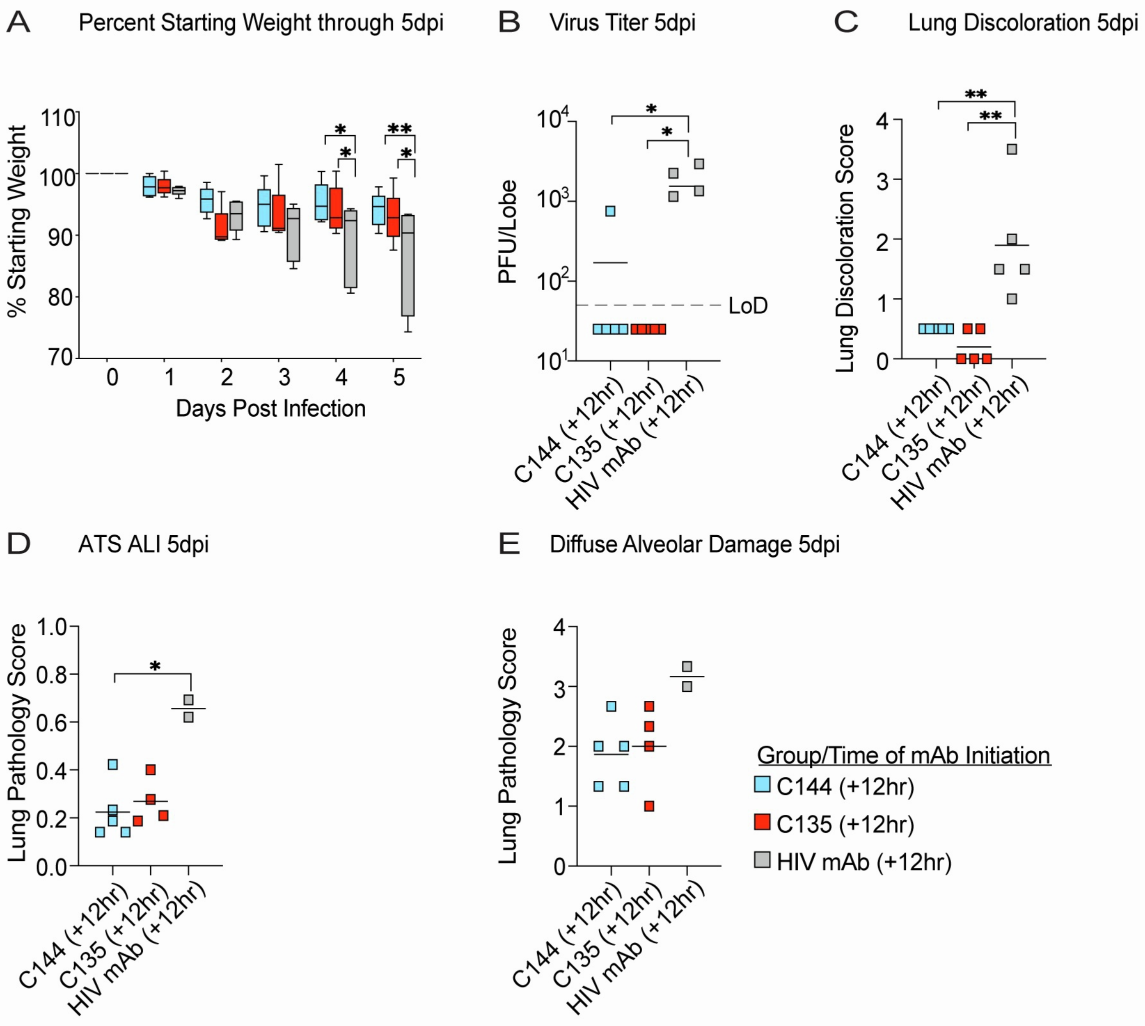

Figure S2. The therapeutic efficacy of mAb monotherapy against SARS-CoV-2 in mice treated at 12 hours post infection.

(A) % starting weight in therapeutically treated mice with C144, C135, or an HIV mAb at 12 hours post infection.

(B) Lung viral titers in therapeutically treated mice at 12 hours post infection.

(C) Lung discoloration score in therapeutically treated mice at 12 hours post infection.

(D-E) Lung pathology in therapeutically treated mice at 12 hours post infection. P values are from a 2-way ANOVA after Sidak's multiple comparisons test. Related to Figure 3.

Figure S3

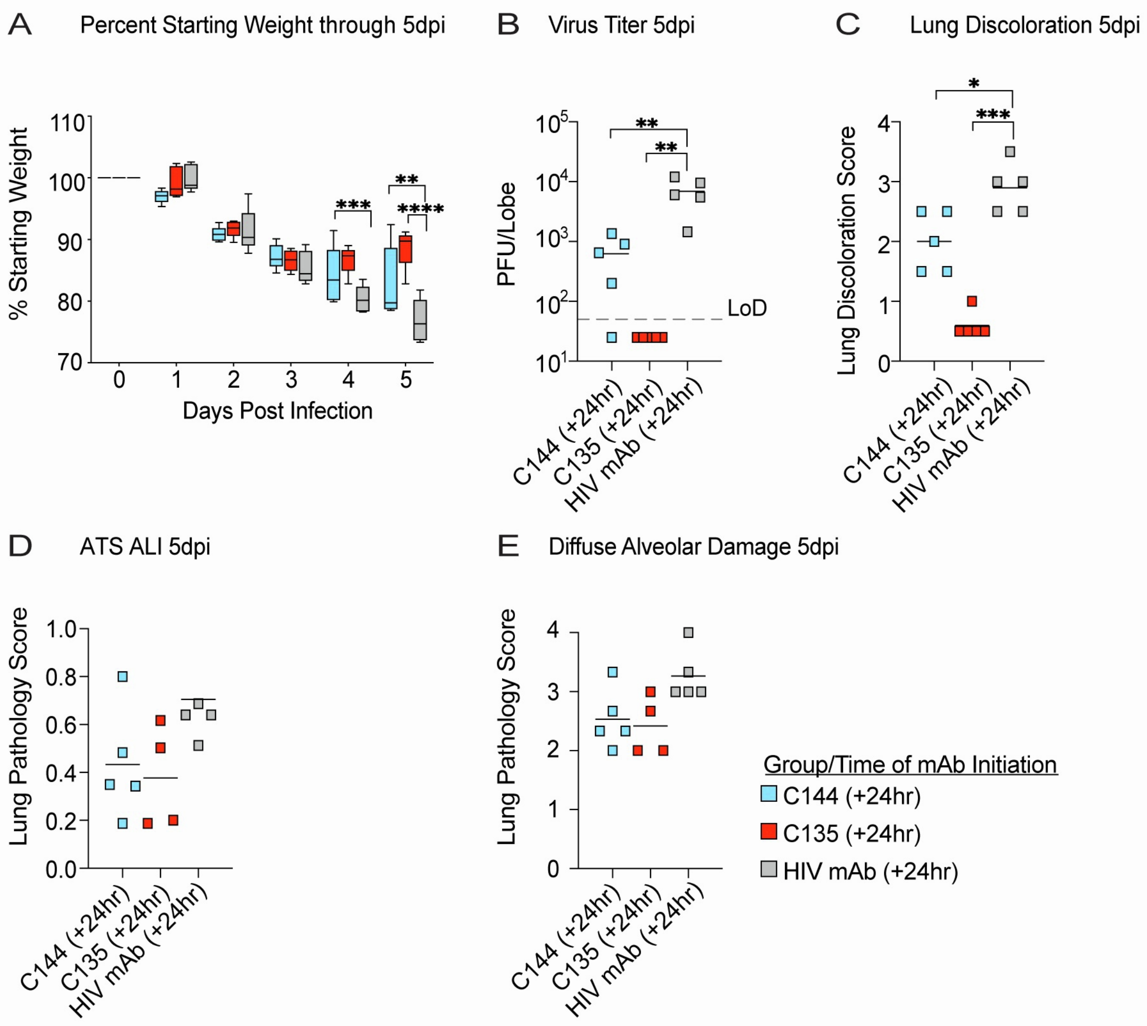

Figure S3. The therapeutic efficacy of mAb monotherapy against SARS-CoV-2 in mice treated at 24 hours post infection.

(A) % starting weight in therapeutically treated mice with C144, C135, or an HIV mAb at 24 hours post infection.

(B) Lung viral titers in therapeutically treated mice at 24 hours post infection.

(C) Lung discoloration score in therapeutically treated mice at 24 hours post infection.

(D-E) Lung pathology in therapeutically treated mice at 24 hours post infection. P values are from a 2-way ANOVA after Sidak’s multiple comparisons test. Related to Figure 3.

Figure S4

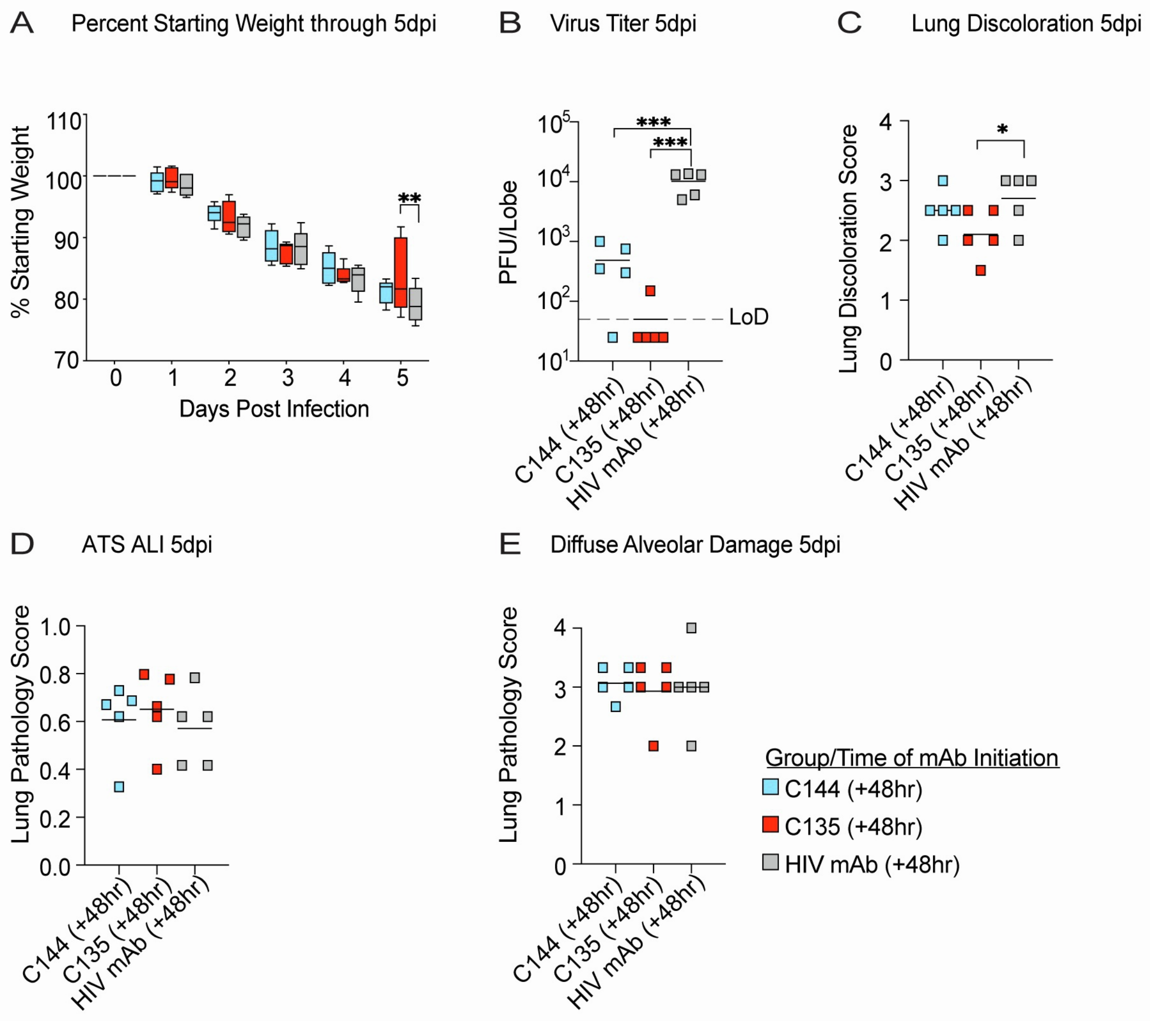

Figure S4. The therapeutic efficacy of mAb monotherapy against SARS-CoV-2 in mice treated at 48 hours post infection.

(A) % starting weight in therapeutically treated mice with C144, C135, or an HIV mAb at 48 hours post infection.

(B) Lung viral titers in therapeutically treated mice at 48 hours post infection.

(C) Lung discoloration score in therapeutically treated mice at 48 hours post infection.

(D-E) Lung pathology in therapeutically treated mice at 48 hours post infection. P values are from a 2-way ANOVA after Sidak's multiple comparisons test. Related to Figure 3.

Figure S5

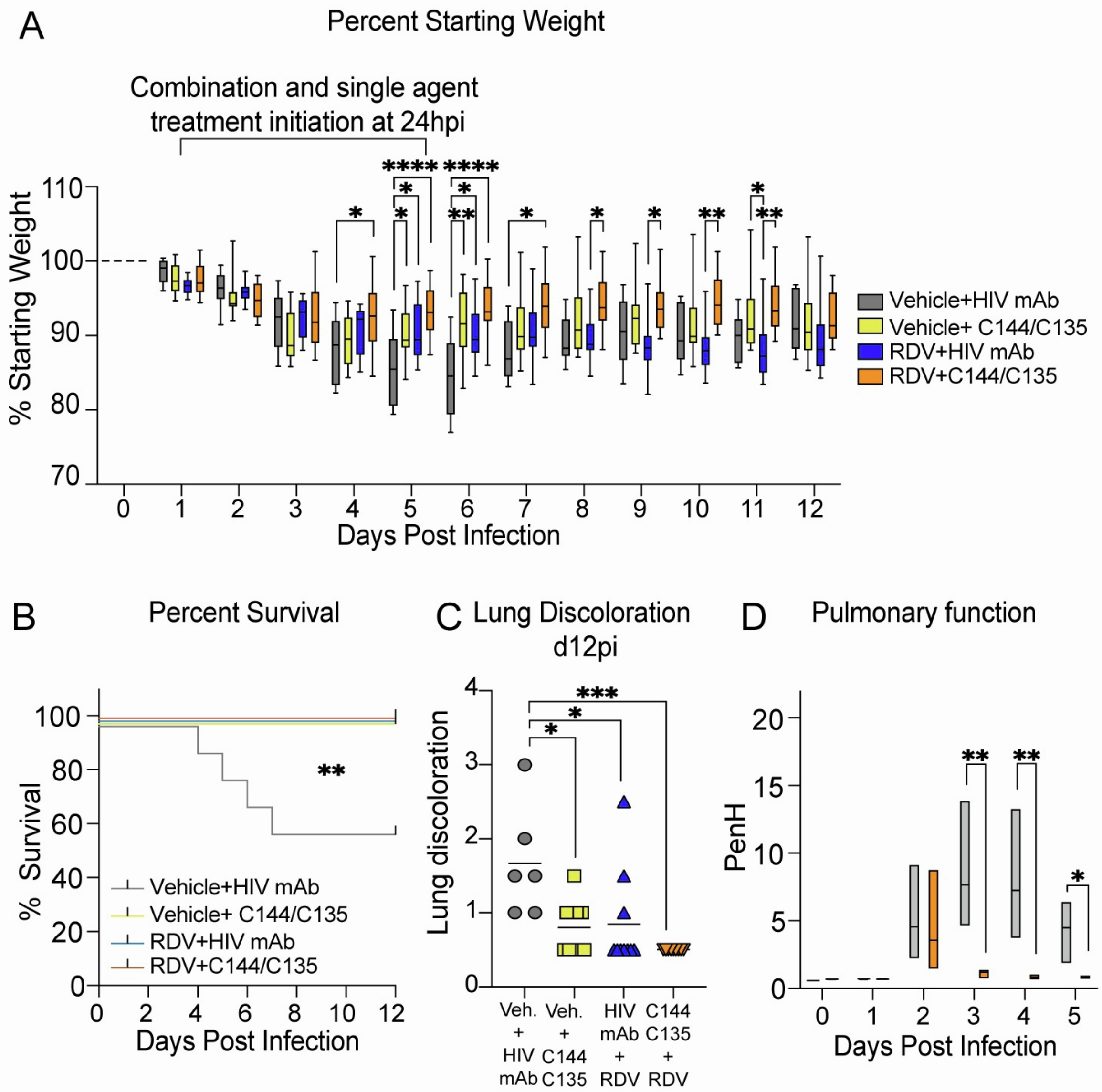

Figure S5. The therapeutic efficacy of RDV and mAbs as single agents and in combination at 24 hours post infection in SARS-CoV-2-infected mice.

(A) % starting weight in therapeutically treated mice with vehicle + HIV mAb, vehicle + C144 + C135, RDV + HIV mAb, and RDV + C144 + C135 at 24 hours post infection through day 12. From left to right, grey bars denote vehicle/control mAb treated mice, yellow bars denote vehicle/mAb therapeutic treatment, blue bars denote RDV/control mAb therapeutic treatment, and orange bars denote RDV/mAb therapeutic treatment.

(B) % mortality in therapeutically treated mice with single agents and combination therapy.

(C) Lung discoloration score in therapeutically treated mice with single agents and combination therapy. "Veh." signifies vehicle treatment.

(D) Pulmonary function in therapeutically treated mice with vehicle + HIV mAb and RDV + C144 + C135. P values are from a 2-way ANOVA after Sidak's multiple comparisons test. Related to Figure 5.
